# A femtosecond time-resolved Bragg coherent diffraction imaging experimental setup at PAL-XFEL for nanoscale lattice dynamics studies

**DOI:** 10.1107/S1600577526007927

**Published:** 2026-10-05

**Authors:** Jinback Kang, Jaeku Park, Chi Hyun Shim, Myung-Hoon Cho, Kook-Jin Moon, Sung Soo Ha, Hyunjung Kim, Sung-Lae Cho, Sunam Kim

**Affiliations:** a XFEL Application Team, Beamline Division, PAL-XFEL, Pohang Accelerator Laboratory, Pohang, Republic of Korea; b Accelerator Control Team, Accelerator Division, PAL-XFEL, Pohang Accelerator Laboratory, Pohang, Republic of Korea; c Center of Ultrafast Phase Transformation, Department of Physics Sogang University Seoul Republic of Korea; d Korea Basic Science Institute Cheongju Chungbuk Republic of Korea; e School of Materials Science and Engineering University of Ulsan Ulsan Republic of Korea; ESRF – The European Synchrotron, France

**Keywords:** X-ray free-electron laser, time-resolved bragg coherent X-ray diffraction imaging, X-ray scattering and spectroscopy beamline, PAL-XFEL

## Abstract

We report on the design and operational characterization of a femtosecond time-resolved Bragg coherent X-ray diffraction imaging (tr-BCDI) setup at the XSS beamline of PAL-XFEL, aimed at enabling direct nanoscale visualization of ultrafast lattice dynamics in individual nanocrystals. The pump–probe temporal resolution was evaluated via time-resolved X-ray diffraction measurements on a Bi (003) epitaxial thin film, confirming a timing precision better than 60 fs through the observation of coherent phonon oscillations. The static coherent imaging capability was benchmarked by reconstructing three-dimensional displacement fields of Au and zeolite nanocrystals, achieving spatial resolutions of 32.3 nm and 50.3 nm, respectively. Crucially, the time-resolved imaging capability was directly demonstrated by tracking the ultrafast structural response of a zeolite nanocrystal under femtosecond laser excitation, successfully capturing the transient shift of the Bragg peak and the corresponding projection of the displacement fields through two-dimensional phase retrieval at a fixed angular condition. These results collectively establish the XSS tr-BCDI setup as a reliable and complementary framework within the global landscape of XFEL facilities, providing direct access to femtosecond-scale structural dynamics in crystalline nanomaterials.

## Abstract 2

The XSS beamline at PAL-XFEL provides a femtosecond time-resolved Bragg coherent X-ray diffraction imaging setup, optimized for tracking transient lattice dynamics and nanoscale strain fields within single crystals during dynamic processes.

## 1. Introduction

The rapid advancement of nanotechnology has created an urgent need to understand the structural dynamics of nanocrystalline materials at atomic length scales and femtosecond timescales. In particular, real-time mapping of lattice strain and structural phase transitions is critical for elucidating the fundamental operating mechanisms of next-generation semiconductors, catalysts, and high-performance energy storage materials (Klimov & McBranch, 1998 ▸; Chen *et al.*, 1996 ▸; Lisiecki *et al.*, 2008 ▸; Strasser *et al.*, 2010 ▸; Giri *et al.*, 2011 ▸; Li *et al.*, 2022 ▸).

Although conventional X-ray diffraction provides only ensemble-averaged structural information, Bragg coherent X-ray diffraction imaging (BCDI) enables three-dimensional visualization of internal strain fields within individual nanocrystals with nanometre-scale spatial resolution (Pfeifer *et al.*, 2006 ▸; Robinson & Harder, 2009 ▸). When combined with the ultrashort, highly coherent pulses delivered by X-ray free-electron lasers (XFELs), BCDI becomes a uniquely powerful approach for probing ultrafast structural dynamics that remain inaccessible with synchrotron sources (Emma *et al.*, 2010 ▸; Ishikawa *et al.*, 2012 ▸; Kang *et al.*, 2017 ▸). However, implementing time-resolved BCDI (tr-BCDI) under pump–probe conditions demands highly sophisticated instrumentation, precise mechanical alignment, and stringent experimental stability, which have collectively limited its broader application (Robinson *et al.*, 2016 ▸).

The PAL-XFEL facility delivers ultrashort, highly coherent, and extremely bright X-ray pulses, providing a unique capability for femtosecond-scale structural investigations. Within this facility, the X-ray Scattering and Spectroscopy (XSS) beamline offers a versatile environment for coherent X-ray scattering across a broad photon energy range of 2.1–15.0 keV (Park *et al.*, 2016 ▸). The self-seeded X-ray source delivers a narrow spectral bandwidth of approximately 500 meV with superior wavelength stability (Nam *et al.*, 2021 ▸), effectively mitigating the stochastic spectral fluctuations inherent to conventional light sources. Combined with highly stable X-ray optics, a synchronized femtosecond pump laser (Eom *et al.*, 2022 ▸), a high-precision diffractometer, and a high-dynamic-range detector (Mozzanica *et al.*, 2014 ▸), the XSS beamline constitutes an optimal environment for the direct visualization of nanoscale lattice dynamics on femtosecond timescales.

Building upon these capabilities, we have developed an experimental setup that fully exploits the coherence, stability, and ultrashort pulse nature of the PAL-XFEL source to enable tr-BCDI. Here we present the design and implementation of this system at the XSS beamline, together with pilot experiments that validate three key aspects of its performance. First, the temporal resolution was characterized through time-zero measurements on a Bi (003) epitaxial thin film, achieving a timing precision better than 60 fs by tracking coherent *A*_1g_ phonon oscillations (Fritz *et al.*, 2007 ▸). Second, the static imaging capability was benchmarked through 3D reconstruction of displacement fields in Au and zeolite nanocrystals, yielding spatial resolutions of 32.3 nm and 50.3 nm, respectively. Finally, the practical adaptability of the setup for dynamic studies was validated through tr-BCDI measurements on a single zeolite nanocrystal. By performing femtosecond laser pump–X-ray probe time-delay scans, we successfully captured the transient changes of the Bragg reflections to track the displacement field evolution, thereby directly unveiling the ultrafast lattice deformations.

## 2. XFEL beam characterization and beamline optics

The self-seeded X-ray beam at PAL-XFEL provides excellent energy stability, providing a critical foundation for successful tr-BCDI measurements. Because individual pulses inherently exhibit shot-to-shot intensity and pointing jitter, all beam parameters reported here represent ensemble averages over 30 shots to ensure statistical stability. The source position is defined as the ‘apparent source’ to account for the combined influence of source dynamics and upstream beamline optics (Vartanyants & Robinson, 2003 ▸). In this section, the three beam properties most critical for tr-BCDI are characterized: intensity stability, longitudinal coherence, and transverse coherence.

To satisfy the spectral coherence requirements for tr-BCDI, the X-ray pulses were spectrally filtered using an Si (111) double-crystal monochromator (DCM) with an energy bandpass of 1.22 eV at 9.0 keV located 31.5 m upstream from the diffractometer center. This configuration effectively suppressed the broadband SASE background while preserving the intrinsic self-seeded bandwidth of 500 meV (Nam *et al.*, 2021 ▸). The filtered spectral width corresponds to a longitudinal coherence length, ξ_L_ = λ^2^/2Δλ, of approximately 1.2 µm, which significantly exceeds the typical dimensions of the investigated nanocrystals and ensures full coherent illumination across the entire sample volume along the longitudinal direction. The DCM-filtered beam maintained a stable average photon flux of 2.57 × 10^11^ to 3.14 × 10^11^ photons per pulse, with shot-to-shot standard deviations of 21.5 to 25.0% across three consecutive measurement days (Fig. S1 of the supporting information), confirming reproducible photon delivery.

The apparent transverse coherence length, ξ_T_ = 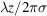, was estimated by determining the effective source-to-sample distance *z* and the full width at half-maximum (FWHM) beam size σ. To evaluate *z*, the angular divergence was experimentally determined by measuring the beam sizes at multiple positions along the beam path. From a weighted linear regression analysis of these measurements (Fig. S2), the beam divergence was found to be 2.76 ± 0.71 µrad horizontally and 4.16 ± 0.94 µrad vertically. Using these divergence values, the apparent photon-source positions relative to the diffractometer center (*z* = 0) were inferred to be *z*_H_ = 103.60 ± 22.72 m upstream horizontally and *z*_V_ = 78.97 ± 14.81 m upstream vertically. This difference of ∼24.6 m constitutes a source astigmatism, which can be reasonably linked to the vertical deflection of the DCM that is expected to shift the apparent source position along the vertical plane (Vartanyants & Robinson, 2003 ▸).

After beam conditioning, the X-rays were focused onto the diffractometer center using Be compound refractive lenses (CRLs) positioned at *z* = 8.1 m, yielding a focal spot size of 9.35 ± 0.35 µm (H) and 10.34 ± 0.73 µm (V) r.m.s. at the sample position (see Section S3 for CRL configuration details). Accounting for the lens magnification and the back-propagation procedure, the effective transverse coherence lengths at the focal plane were evaluated to be 742 ± 28 nm in the horizontal direction and 930 ± 65 nm in the vertical direction. These coherence lengths exceed the ∼500 nm dimensions of the investigated nanocrystals, ensuring full coherent illumination across the entire crystal volume necessary for reliable BCDI reconstruction.

## 3. Instrumentation and alignment procedures for tr-BCDI

The instrumentation for tr-BCDI is a highly integrated system that combines a femtosecond pump laser, an XFEL source, X-ray optics, diagnostics, a high-precision four-circle diffractometer, and an X-ray detector. The XFEL source, pump laser, and diagnostic modules are temporally synchronized, with their detailed specifications described elsewhere (Kang *et al.*, 2017 ▸). In this section, the components directly relevant to tr-BCDI measurement are focused on: the four-circle diffractometer, and the procedures required to accurately align the pump laser, the focused XFEL beam, and a single nanocrystal within a common interaction volume.

**Figure 1:**
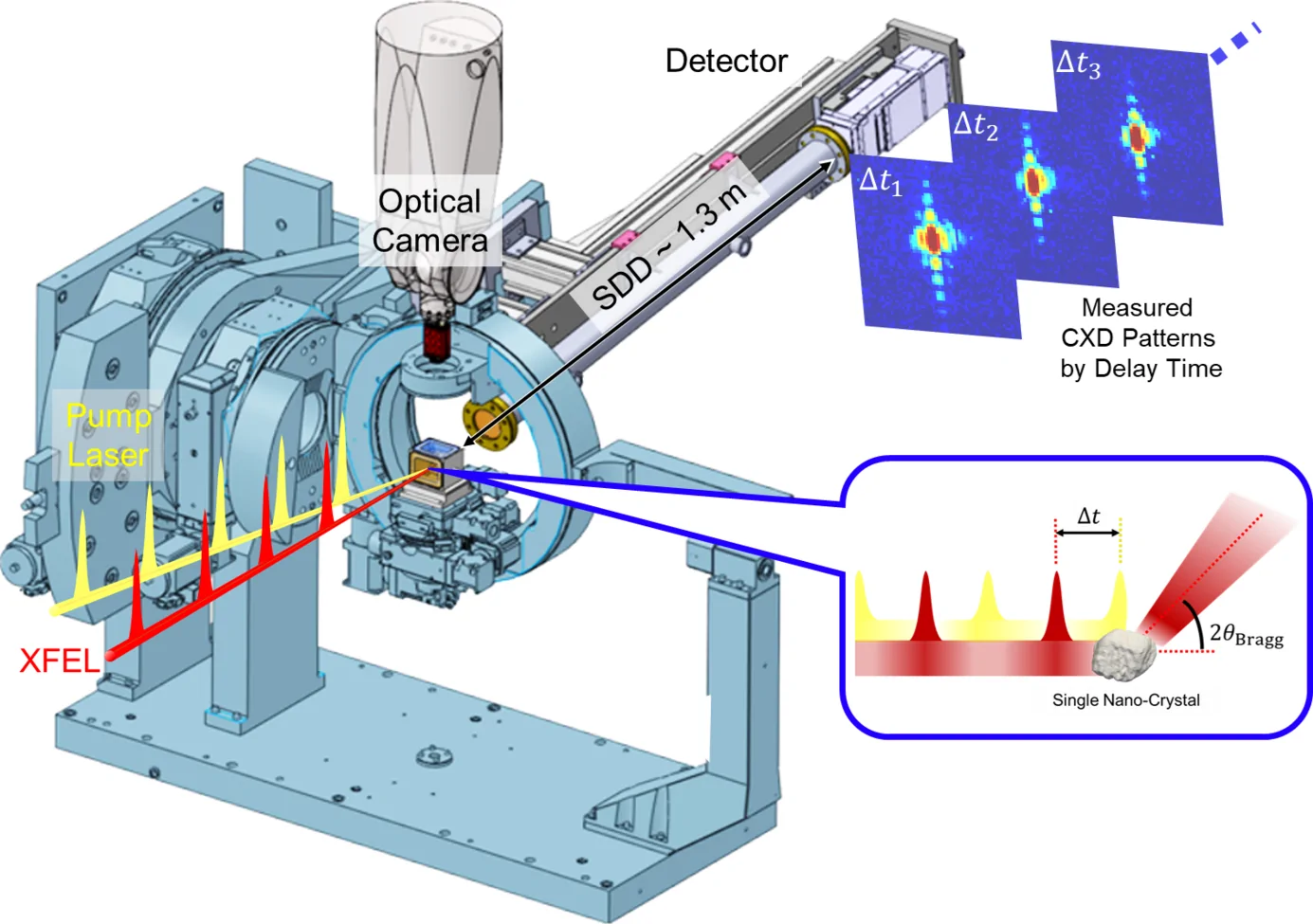
Schematic layout of the tr-BCDI setup integrated with a four-circle diffractometer. In the pump–probe sequence, the XFEL pulse was subsequently delivered after the pump laser at the interaction point (IP). The XFEL beam direction remained fixed, whereas the pump laser incidence could be configured horizontally or vertically with user’s request. A vertically oriented optical camera is positioned above the sample stage for precise alignment, ensuring that the center of rotation of the diffractometer is perfectly coincident with the spatial IP of the XFEL and the pump laser.

Laser-pump/X-ray-probe tr-BCDI experiments are conducted using a Huber four-circle diffractometer (θ, 2θ, φ, χ) with an angular precision of approximately 7 × 10^−4^ degrees, equipped with a three-axis piezo-driven linear stage providing approximately 30 nm positioning precision. Fig. 1 ▸ shows a schematic layout of the configuration. In this pump–probe scheme, a femtosecond laser pulse excites the nanocrystal to induce transient lattice strain, and after a controlled time delay Δ*t* an XFEL probe pulse illuminates the sample to generate coherent X-ray diffraction (CXD) patterns. The X-ray detector is positioned at the corresponding 2θ angle determined by the lattice spacing of the nanocrystal and the 9.0 keV X-ray wavelength, in accordance with Bragg’s law.

The diffractometer supports a detector arm travel range of 0–90° in 2θ, accommodating a wide range of Bragg reflections accessible within the XSS photon energy range of 2.1–15.0 keV. Scattered X-rays travel through a flight tube maintained under rough vacuum (evacuated by a rotary vane pump) to suppress the air scattering background between the sample and the detector. Diffraction patterns are recorded using a 0.5 M pixel JUNGFRAU detector (Mozzanica *et al.*, 2014 ▸), a charge-integrating hybrid pixel detector comprising 512 × 1024 pixel array with a pixel size of 75 µm × 75 µm, a dynamic range of up to 10000 photons pulse^−1^ pixel^−1^ at 12 keV, and single-photon sensitivity.

The minimum oversampling ratio required for reliable phase retrieval in CDI is 2 (Sayre, 1980 ▸; Miao *et al.*, 1998 ▸). Under the experimental conditions employed in this work [photon energy of 9.0 keV, sample-to-detector distance (SDD) of 1.3 m and pixel size 75 µm], the maximum nanocrystal size satisfying an oversampling ratio of 2 is given by *D* ≤ λSDD/(2*p*) ≃ 1.19 µm, where λ = 1.378 Å is the X-ray wavelength and *p* is the pixel size. The nanocrystals studied in this work (300–500 nm) are well within this limit, ensuring adequate oversampling of the coherent diffraction patterns for accurate 3D phase retrieval.

The spatial alignment procedure begins by centering a reference pin at the rotation center of the diffractometer using a optical camera. The entire diffractometer is then translated so that the pin intersects the XFEL beam path, providing a coarse alignment. Subsequently, the pin is removed and a GaAs wafer is positioned at the sample location to enable fine alignment. To precisely match the rotation center of the diffractometer to the XFEL beam, the height of either the entire diffractometer or the wafer surface is incrementally adjusted relative to the beam while performing θ and χ axis rocking scans. The positional stability of the X-ray footprint is monitored during these scans: a stationary footprint centroid indicates proper alignment, whereas any wobbling signifies spatial misalignment. In such cases, the height is iteratively refined until the footprint wobble is minimized, ensuring accurate coincidence between the beam and the rotation center. Finally, the pump laser is steered to coincide with this established position, ensuring that both beams form a single, synchronized interaction volume at the sample position.

## 4. Pump–probe timing characterization and tr-BCDI performance

### 4.1. Validation of pump–probe timing

**Figure 2:**
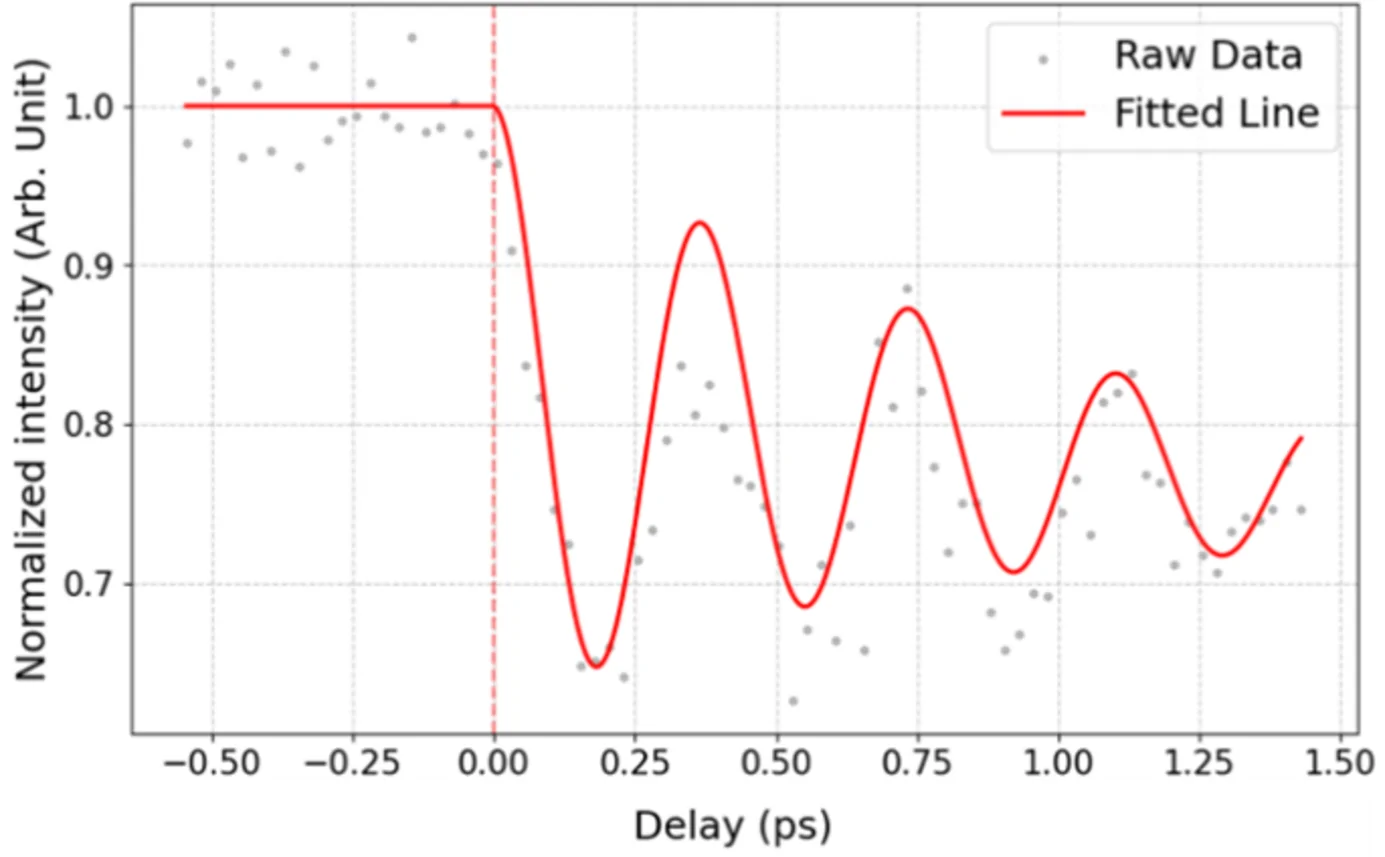
Time-resolved evolution of the Bi (003) Bragg peak intensity with its corresponding fitting curve. Data were acquired using a 266 nm optical pump (2 mJ cm^−2^) and a CRL-focused 9.0 keV XFEL probe (∼30 fs). The fit yields a coherent phonon frequency of 2.71 T Hz, demonstrating an experimental timing precision of <60 fs. The extracted best-fit parameters are as follows: instrument response width = 0.0995 ps, oscillatory amplitude = 0.1902, coherent phonon frequency = 2.7095 THz, and decay lifetime = 0.8570 ps.

The temporal resolution of the pump–probe setup was validated by characterizing the ultrafast lattice dynamics of a 40 nm-thick epitaxial Bi (003) thin film grown on a Si (111) substrate with a 3 nm GaSb buffer layer. Optical pump pulses at a wavelength of 266 nm—with a fluence of 2 mJ cm^−2^ and a beam size of approximately 200 µm (FWHM) under normal incidence—excited the sample below the radiation damage threshold, while delayed X-ray pulses probed the resulting structural response. The X-ray probe pulses with a photon energy of 9.0 keV and a nominal pulse duration of approximately 30 fs (Nam *et al.*, 2021 ▸) were focused onto the sample using a Be CRL to a spot size of approximately 20 µm (FWHM). This configuration ensured that the optically pumped region was significantly larger than the probed area, allowing the measured dynamics to reflect a uniformly excited volume. The temporal evolution of the Bi (003) Bragg peak intensity, recorded with a 25 fs step size, exhibited clear oscillatory modulation originating from the coherent *A*_1g_ phonon mode. Fitting this oscillation yielded an instrumental time resolution better than 60 fs, and the extracted phonon frequency of 2.71 THz agreed well with previously reported values (Fritz *et al.*, 2007 ▸), confirming the precision of the instrumental time resolution. The time-dependent Bi (003) Bragg peak intensity and the corresponding fit are shown in Fig. 2 ▸.

### 4.2. Demonstration of BCDI on nanocrystals

Building on this temporal validation, we next examined the coherent imaging capability of the setup using Au and zeolite nanocrystals, which serve as complementary test samples with contrasting scattering strengths and structural characteristics. The Au nanocrystals, produced via high-temperature solid state dewetting (Heyraud & Metois, 1980 ▸), provided strong scattering contrast and well defined facets, enabling high-resolution benchmarking. The zeolite nanocrystals, synthesized through a two-step hydro­thermal crystallization process (Chao *et al.*, 1986 ▸; Gao *et al.*, 2004 ▸), offered a complementary test case due to their weaker scattering and porous internal framework. For both samples, a 9.0 keV self-seeded X-ray beam focused by Be CRLs to approximately 20 µm (FWHM) at the sample position was used with an SDD of 1.3 m.

**Figure 3:**
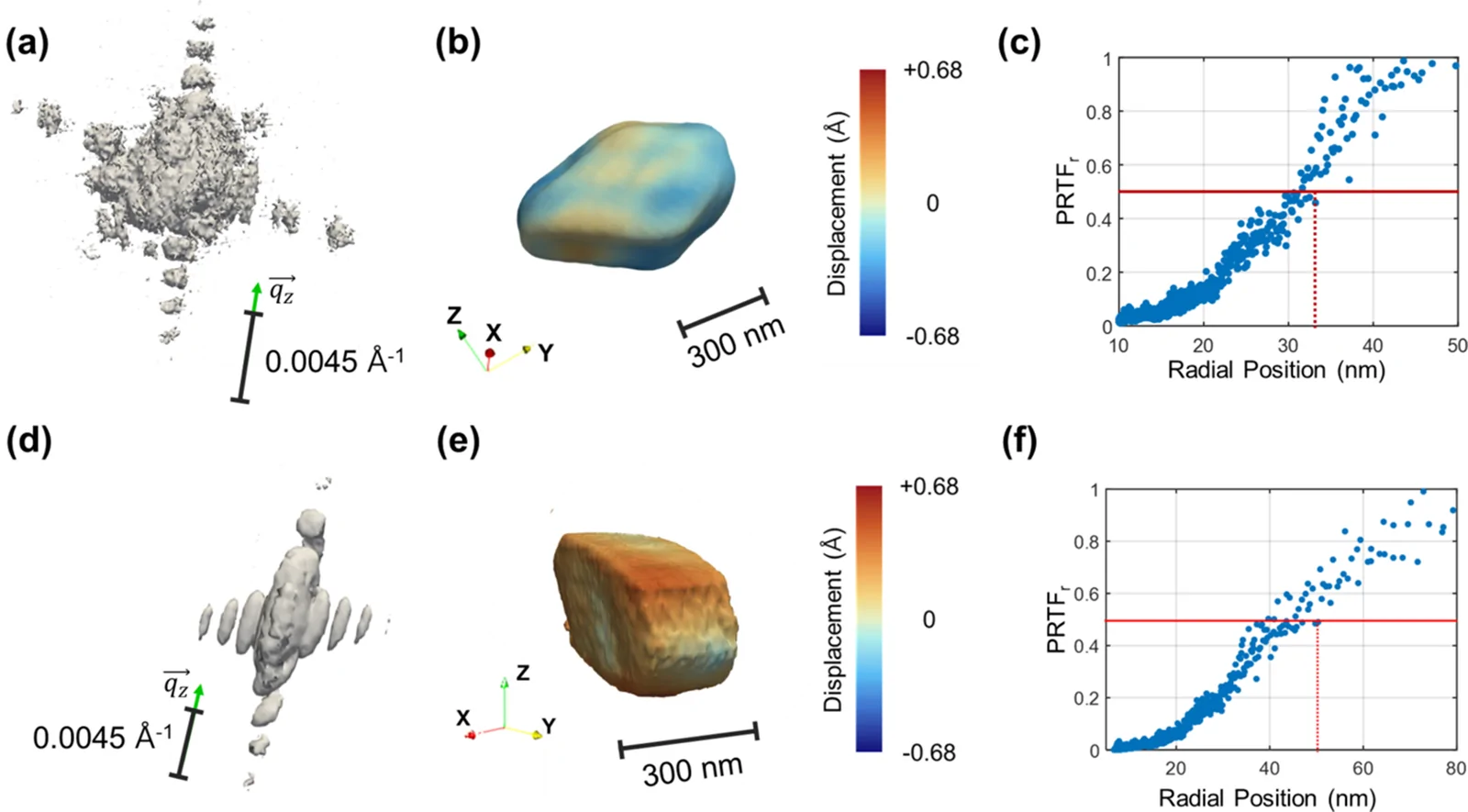
BCDI results of Au and zeolite nanocrystals. (*a*) 3D CXD pattern of Au nanocrystal at the Au (111) Bragg reflection (9.0 keV). (*b*) 3D reconstructed displacement field of the Au nanocrystal. (*c*) PRTF; spatial resolution 32.3 nm (PRTF = 0.5 criterion). (*d*) 3D CXD pattern of zeolite nanocrystal at the (020) Bragg reflection. (*e*) 3D reconstructed displacement field. (*f*) PRTF; spatial resolution 50.3 nm.

To ensure statistical stability against the inherent shot-to-shot intensity fluctuations of the XFEL, the CXD patterns from a single Au nanocrystal with a particle size of approximately 600 nm were recorded at the (111) Bragg reflection. The individual profiles were accumulated over 30 pulses per angular step, corresponding to an effective exposure time of 0.5 s at a 60 Hz repetition rate (see Section S5 for a detailed justification of the accumulation number). The resulting patterns were reconstructed using a consistent phase retrieval workflow (detailed in Section S4). To quantify the reconstruction quality, the spatial resolution was evaluated via phase retrieval transfer function (PRTF) analysis (Shapiro *et al.*, 2005 ▸; Chapman *et al.*, 2006 ▸), yielding a resolution of 32.3 nm [Figs. 3 ▸(*a*)–3(*c*)].

Following the benchmarking with Au, the experimental capabilities of the setup were further demonstrated using zeolite nanocrystals (∼500 nm in diameter). The CXD patterns were recorded at the (020) Bragg reflection, and the reconstructed 3D strain field achieved a spatial resolution of 50.3 nm, as quantified by the PRTF analysis [Figs. 3 ▸(*d*)–3(*f*)]. Having established the static imaging performance of the setup, we next sought to demonstrate its practical capability under dynamic conditions through tr-BCDI measurements.

### 4.3. Time-resolved BCDI demonstration

**Figure 4:**
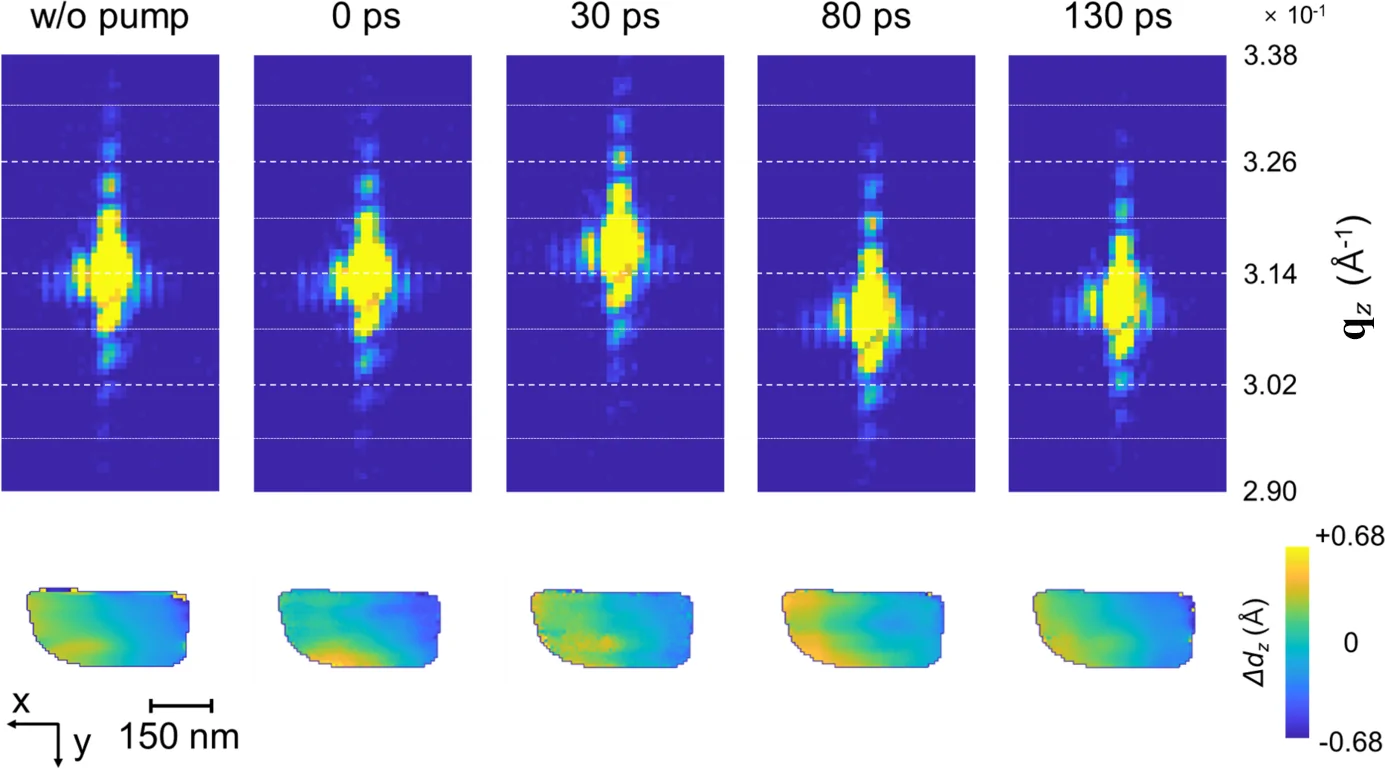
Temporal evolution of the zeolite (020) Bragg reflection and the corresponding retrieved displacement fields. A comparative analysis is presented for two conditions: without optical pump laser excitation and with optical pump laser excitation (nominal fluence of 2 mJ cm^−2^) at delay times of 0, 30, 80, and 130 ps (from left to right). The upper row within each condition displays the 2D CXD profiles centered on the (020) Bragg peak (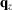 = 3.14 × 10^−1^ Å^−1^), where the white grid lines indicate a momentum transfer. The lower row presents the corresponding 2D phase maps, showing the displacement fields projected along the [020] Bragg vector.

To evaluate the time-resolved imaging capability of the setup under dynamic conditions, the ultrafast structural response of the zeolite nanocrystal (020) Bragg reflection was tracked using the 266 nm femtosecond pump laser (Fig. 4 ▸). The optical pump pulses were delivered with a nominal fluence of 2 mJ cm^−2^, ensuring sufficient structural excitation while avoiding radiation damage. 2D phase retrieval was performed directly on the individual CXD patterns, yielding displacement fields projected along the [020] Bragg vector.

Upon laser excitation, the positions of the Bragg peaks exhibited a distinct structural response at a delay time of 30 ps, initially shifting toward a higher momentum transfer (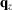) before reversing toward a lower **q** and undergoing subsequent thermal relaxation by 130 ps. This behavior suggests a sequence of nanoscale lattice dynamics involving an initial rapid structural contraction followed by thermal expansion induced by the optical pump. These observations provide a compelling demonstration of the capability of the developed setup for stable tr-BCDI measurements. Furthermore, by successfully capturing these transient structural features, this work demonstrates the practical feasibility of ultrafast pump–probe BCDI investigations, thereby establishing a robust framework for tracking transient lattice deformations within functional nanocrystals at extremely short timescales.

## 5. Summary and conclusion

In summary, we have successfully developed and characterized a femtosecond tr-BCDI experimental setup at the PAL-XFEL XSS beamline, optimized for capturing transient lattice dynamics at the nanoscale. Comprehensive measurements validated the high spatial coherence of the focused X-ray beam, while a systematic frame-dependence analysis verified that a 30-frame accumulation scheme effectively mitigates stochastic pulse variations, yielding spatial resolutions of 32.3 nm and 50.3 nm for Au and zeolite nanocrystals, respectively. The temporal resolution of the integrated laser-pump and X-ray-probe system was confirmed to be better than 60 fs, as shown by the high-fidelity tracking of coherent *A*_1g_ phonon oscillations of Bi (003) thin film. We successfully captured the transient lattice dynamics of a single zeolite nanocrystal under femtosecond laser excitation, demonstrating the robust operation of both the optical synchronization and the phase-retrieval workflow under dynamic conditions. This established tr-BCDI setup opens up new avenues for investigating structural phase transitions, acoustic phonon propagation, and mechanical strain evolution within individual photo-active nanocrystals and functional nanostructures on ultrafast timescales.

## 6. Related literature

The following references, not cited in the main body of the paper, have been cited in the supporting information: Fienup (1982 ▸); Li & Zhou (2017 ▸).

## Supplementary Material

Sections S1 to S5 including Figs. S1 to S5. DOI: 10.1107/S1600577526007927/ok5167sup1.pdf

## Data Availability

The raw XFEL diffraction data, reconstructed Bragg coherent diffraction imaging (BCDI) volumes, and analysis scripts supporting the results of this study are stored on the PAL-XFEL data server. Due to storage and access restrictions, these data are available from the corresponding author upon reasonable request.
