# Supplementary material for: A femtosecond time-resolved Bragg coherent diffraction imaging experimental setup at PAL-XFEL for nanoscale lattice dynamics studies
Source: J Synchrotron Radiat. 2026 Oct 5;33(Pt 6):1814–9. doi: 10.1107/S1600577526007927 (PMC13753867; doi:10.1107/S1600577526007927)
Supplement: Supplementary file 1 [file s-33-01814-sup1.pdf]

JOURNAL OF SYNCHROTRON RADIATION

Volume 33 (2026)

Supporting information for article:

A femtosecond time-resolved Bragg coherent diffraction imaging experimental setup at PAL-XFEL for nanoscale lattice dynamics studies

Jinback Kang, Jaeku Park, Chi Hyun Shim, Myung-Hoon Cho, Kook-Jin Moon, Sung Soo Ha, Hyunjung Kim, Sung-Lae Cho and Sunam Kim

S1. X-ray Beam Intensity Monitoring and Stability

To ensure the reliability of the tr-BCDI measurements, the stability of the X-ray photon flux was continuously monitored throughout the experimental period. The quadrant beam position monitor (QBPM), positioned 20.1 m upstream from the diffractometer center and following the DCM, provided a linear and quantitative measure of the delivered photon flux.

As shown in Figure S1, the filtered X-ray pulses maintained a consistent average intensity and a stable shot-to-shot distribution. The data captured at identical time intervals (23:30 KST) over three different days (Dec 9, 11, and 13) demonstrate the operational robustness of the PAL-XFEL self-seeding mode and the DCM optics. The observed intensity fluctuations, characterized by standard deviation (σ), remained within the expected range for stable data acquisition, supporting the validity of the CXD patterns used for phase retrieval.

S2. Characterization of Focused X-ray Beam Properties and Transverse Source Astigmatism

To accurately evaluate the spatial coherence at the sample position, the angular divergence and spatial propagation properties of the X-ray beam were experimentally characterized. As shown in the beamline diagnostic configurations (Figure S2), the transverse beam dimensions were measured at three distinct locations along the beam path: the optical hutch (OH), the X-ray scattering and spectroscopy (XSS) beamline, and the nano-crystallography and coherent imaging (NCI) beamline. Relative to the diffractometer center at XSS (z = 0), these measurement points within the respective stations were located at approximately z = 27.5 m (OH), z = 9.3 m (XSS), and z = –8.1 m (NCI), representing positions upstream and downstream, respectively. The dimensions were extracted using a series of slit scans, where the integrated intensity for each step was recorded by the quadrant beam position monitors (QBPMs) at each station. To ensure statistical reliability, the scan procedure was repeated four times for both the horizontal and vertical directions.

The longitudinal evolution of the full width at half maximum (FWHM) beam size $\sigma_{FWHM}(z)$ was modeled using a least-squares regression analysis based on the geometric expansion of the beam profile away from the apparent source position, defined by the following hyperbolic relation:

$$\sigma_{FWHM}(z) = \sqrt{s_{FWHM}{}^2 + (z - z_0)^2 \cdot \Delta\theta_{FWHM}{}^2}$$

where s_{FWHM} is the effective FWHM photon-source size, $\Delta\theta_{FWHM}$ is the FWHM beam divergence, and z_0 is the effective source position. By performing this regression analysis on the experimentally measured data points, the apparent beam divergence was determined to be 2.76 ± 0.71 μrad in the horizontal direction and 4.16 ± 0.94 μrad in the vertical direction. Concurrently, the calculated apparent photon-source positions exhibited a distinct mismatch between the horizontal (at z_H =

103.60 ± 22.72 m) and vertical (at z_V = 78.97 ± 14.81 m) planes, revealing a pronounced transverse source astigmatism of approximately 24.6 m.

This discrepancy between the horizontal and vertical source positions, constituting a source astigmatism of approximately 24.6 m, is likely attributable to a combination of several factors inherent to the beamline configuration. Optical imperfections in the beamline components — including wavefront aberrations introduced by the Be CRLs and imperfection of the DCM crystals — may further contribute to the observed astigmatism. Shot-to-shot jitter of the XFEL source, including pointing instability may also introduce systematic biases into the divergence measurements when averaged over a finite number of pulses. A comprehensive quantitative understanding of the source astigmatism will require dedicated beamline optical ray-tracing simulations and systematic experimental investigations under controlled conditions, which are planned as part of future beamline optimization efforts.

S3. Characterization of Focal Spot and Transverse Coherence Length

To ensure optimal beam focusing and to verify the spatial coherence length, the focal spot size was characterized at the rotation center of the diffractometer. The transverse dimensions of the focused X-ray beam were determined by performing translation scans of a single Au nanocrystal with lateral dimensions of approximately 300 nm × 300 nm across the beam focal plane in both horizontal and vertical directions. In this procedure, the physical size of the nanocrystal served as an effective instrumental resolution function (IRF), allowing the beam profile to be extracted from the measured interaction volume. At each scan position, 30 consecutive XFEL shots were averaged to obtain a stable region-of-interest (ROI)–integrated intensity of the coherent X-ray diffraction pattern of the Au nanocrystal, from which the beam-interaction profile was constructed.

As shown in Figures S4(a) and S4(b), scattering intensity profiles were recorded while the nanocrystal was incrementally translated through the focused beam. The horizontal scan followed a transverse trajectory directly across the focal plane, whereas the vertical scan was performed along a path tilted by θ = 13.4° relative to the beam axis, consistent with the Au (111) Bragg reflection geometry. For the vertically tilted scan, the recorded motor positions were geometrically transformed to recover the true transverse beam dimensions relative to the beam axis. To extract the intrinsic beam profile, the measured intensity distributions were deconvolved with the IRF.

These high-precision measurements at the sample position yielded intrinsic focal beam sizes (σ_{focus}) of 9.35 ± 0.35 μm in the horizontal direction and 10.34 ± 0.73 μm in the vertical direction in terms of root-mean-square (rms) standard deviation, which corresponds to full width at half maximum (FWHM) values of 22.03 ± 0.82 μm and 24.35 ± 1.72 μm, respectively. Following the optical propagation framework (Vartanyants & Robinson, 2003), the effective photon-source sizes (σ_{source}) at the apparent source planes were derived by back-propagating these focal dimensions while

accounting for the geometric magnification factor (M_H = 0.085 ± 0.020, M_V = 0.114 ± 0.024) of the Be compound refractive lens (CRL) system via the relation $\sigma_{\mathrm{source}} = \sigma_{\mathrm{focus}}/M$. This back-propagation procedure yielded derived rms photon-source sizes of 110.3 ± 26.6 μm horizontally and 90.5 ± 20.0 μm vertically.

As defined in the main text, the transverse coherence lengths at the IP, ξ_H and ξ_V, are calculated using the measured values and beam parameters (z_H = 103.60 m and z_V = 78.97 m, λ = 1.378 Å) as follows: ξ_H = 742 ± 28 nm and ξ_V = 930 ± 65 nm. The consistency between these experimentally derived coherence parameters and the stable high-contrast Bragg fringes observed experimentally validates both the robust performance of the focusing optics and the accuracy of the spatial coherence lengths utilized for the tr-BCDI reconstructions.

S4. Phase-Retrieval Workflow and Three-dimensional Reconstruction Protocols

The three-dimensional (3D) coherent X-ray diffraction (CXD) patterns of the nanocrystals were systematically reconstructed using a phase-retrieval workflow. For the static benchmarking of the gold (Au) nanocrystals, rocking curve measurements were performed around the Au (111) Bragg angle (2θ = 31º, θ = 13.46º), scanning the θ angle over 81 steps with an angular increment of 7.5 × 10^{-3} degrees. Complementarily, the zeolite nanocrystals were characterized at the (020) Bragg angle (2θ = 7.89º, θ = 4.1º), with the corresponding rocking curve acquired over 101 steps with an angular increment of 7.5 × 10^{-3} degrees. To ensure sufficient signal-to-noise ratios and effectively suppress stochastic intensity fluctuations, each individual diffraction frame was recorded by accumulating 30 X-ray pulses, corresponding to an effective exposure time of 0.5 seconds per angular step (as justified in Supplementary Section S5).

The phase-retrieval process for both nanocrystal systems employed a consistent algorithmic sequence to guarantee the precision and reproducibility of the reconstructed complex wavefields. Each independent reconstruction run comprised a total of 850 iterations, structured into four repeating blocks consisting of 20 iterations of error reduction (ER) (Fienup, 1982) followed by 180 iterations of relaxed averaged alternating reflections (RAAR, with a relaxation parameter of β = 0.9) (Li & Zhou, 2017). The overall sequence was finalized with an additional 50 iterations of ER to ensure optimal numerical convergence at the final state.

During the iterative process, the physical boundary of the object support was dynamically updated using the shrink-wrap algorithm based on a Gaussian-blurred thresholding mechanism. To mitigate numerical stagnation within local minima and broaden the solution search space, a Guided-CDI approach was implemented using a population size of 10 candidates tracked across 5 successive generations. The final real-space density and phase maps were obtained by averaging the 5 best-performing candidates (the averaged-5 return) from the final generation. This rigorous workflow

yielded highly robust and reproducible visualizations of the internal lattice strain and displacement fields within the individual nanocrystals.

S5. Justification of 30-Shot Accumulation

The choice of a 30-shot accumulation was systematically optimized by evaluating the convergence behavior of the achievable spatial resolution as a function of the number of accumulation frames (N). The estimated spatial resolution at each value of N was defined as $2\pi/\Delta\overrightarrow{q_{\max}}$, where $\Delta\overrightarrow{q_{\max}}$ denotes the maximum accessible momentum transfer at which a meaningful single-photon signal remains distinctly detectable above the background noise level. In practice, however, the implementation of data averaging and normalization strictly with respect to the number of frames (N) inherently bounds the high-$\vec{q}$ signals, causing the observable $\Delta\overrightarrow{q_{\max}}$ to systematically converge. It is worth noting that a rigorous pulse-by-pulse normalization against the total incident beam intensity is theoretically and practically unfeasible here, because the sub-micron crystal intercepts only a tiny fraction of the overall beam profile, a fraction that fluctuates randomly due to the inherent spatial pointing jitter of the XFEL. For each value of N, ten independent N-shot averaged CXD frames were constructed from non-overlapping subsets of the acquired data to ensure statistical robustness and quantify reproducibility—for example, the data point at N = 25 represents ten independently averaged frames, each obtained by averaging 25 individual single-shot frames. This systematic evaluation was performed on the Au (111) Bragg reflection across a wide range of N, yielding the statistical outcomes plotted in Figure S5.

At N = 1, the estimated spatial resolution is highly constrained (~78.3 nm) and exhibits a remarkably large statistical uncertainty. This limitation stems directly from the intrinsic shot-to-shot intensity fluctuations and spatial pointing jitter characteristic of individual XFEL pulses. As the shot accumulation number increases, the estimated spatial resolution improves substantially, and the statistical error bars contract significantly, reflecting the effective suppression of stochastic noise.

Crucially, the resolution curve exhibits a distinct diminishing-returns behavior, entering a stable plateau regime near N = 30, where the estimated resolution stabilizes at approximately 30 nm. Beyond this threshold of N = 30, accumulating additional shots yields no meaningful improvement in the accessible $\Delta\overrightarrow{q_{\max}}$ or error reduction, while severely wasting valuable beamtime that could otherwise be allocated to expanding the time-delay points in the pump–probe scan. These quantitative findings demonstrate that the empirical selection of N = 30 resides immediately at this physical plateau. Therefore, N = 30 represents a statistically verified balance that maximizes measurement reproducibility and spatial resolution while maintaining optimal experimental efficiency for tr-BCDI measurements.

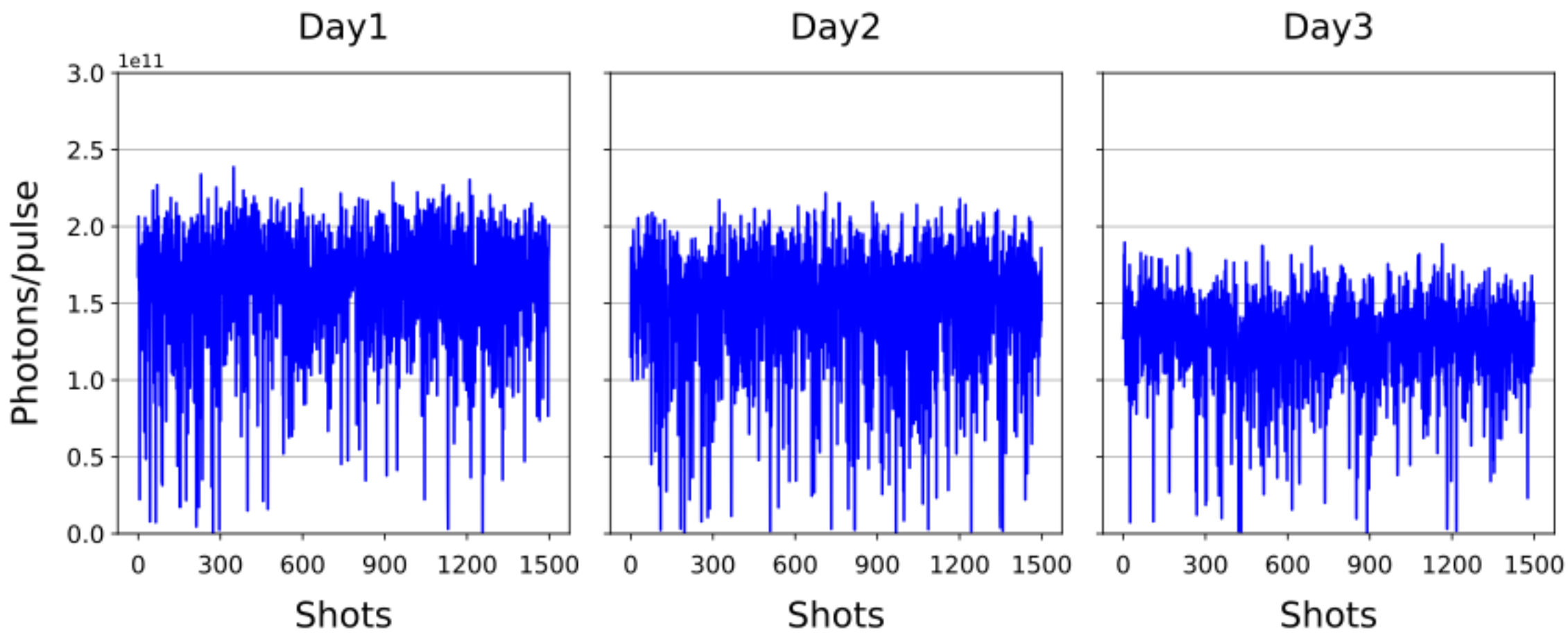

Figure S1 Shot-to-shot stability of the DCM-filtered X-ray photon flux. Representative photon flux distributions were recorded by the QBPM downstream of the DCM at 23:30 (KST) on December 9, 11, and 13, 2025. Each panel shows 1,500 consecutive shots acquired at a repetition rate of 60 Hz. The measured photon flux: Day 1 – mean: 3.14×10^{11}, std (standard deviation): 7.07×10^{10} (22.5%); Day 2 – mean: 2.57×10^{11}, std: 6.42×10^{10} (25.0%); Day 3 – mean: 2.73×10^{11}, std: 5.86×10^{10} (21.5%).

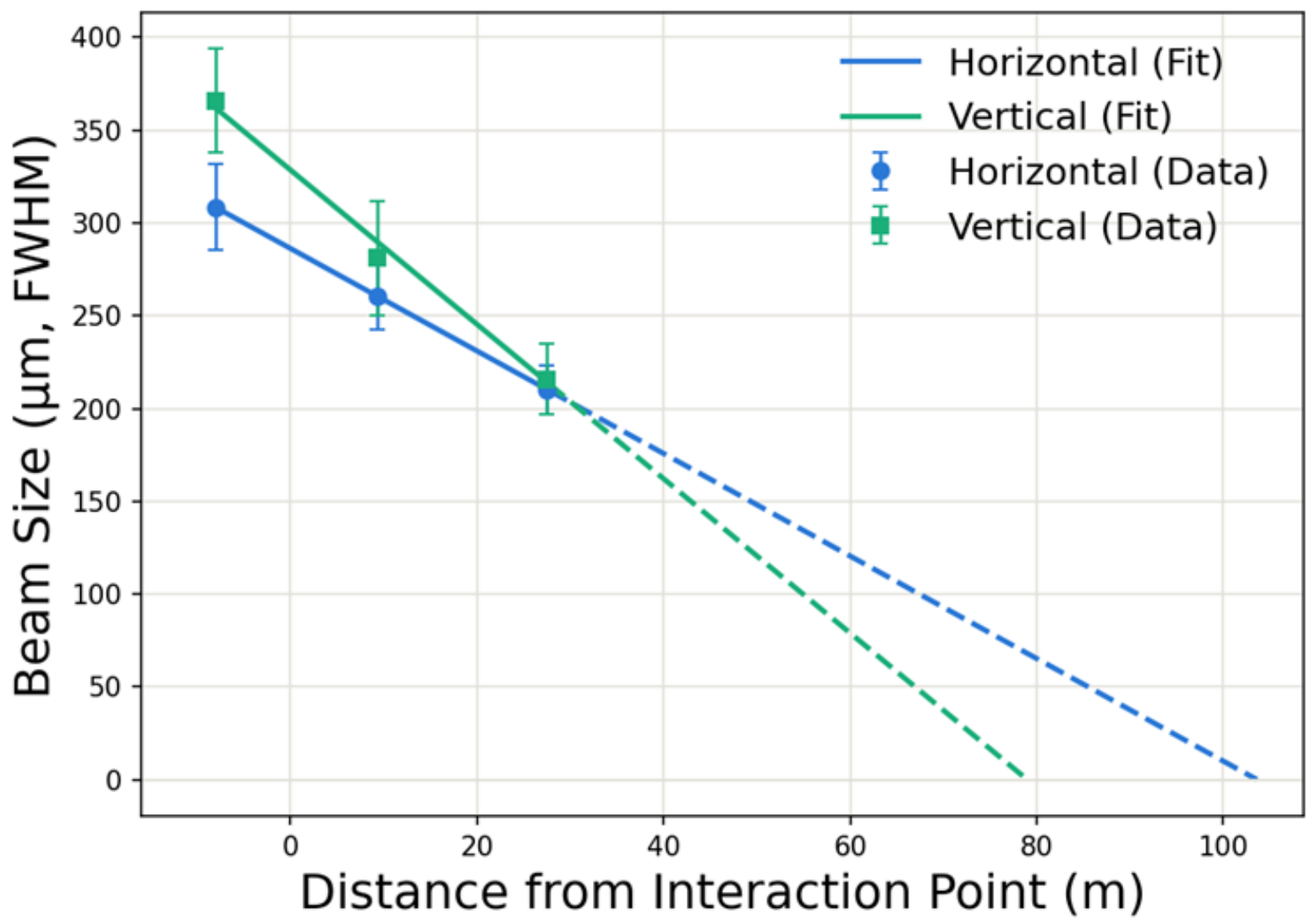

Figure S2 Beam propagation and source characterization. Transverse beam size (FWHM) as a function of distance relative to the IP (z = 0). The beam dimensions were measured at three distinct locations along the beamline, with the coordinate system defined such that the negative and positive directions represent the downstream and upstream positions, respectively. Each data point represents the average of four independent slit scans, with error bars indicating the root mean square (rms) fluctuation. Linear fits to the measured beam sizes were used to determine the apparent beam divergence and the effective source positions for both the horizontal and vertical planes. The longitudinal separation between the horizontal and vertical intercepts on the distance axis quantifies the source astigmatism induced by the beamline optics.

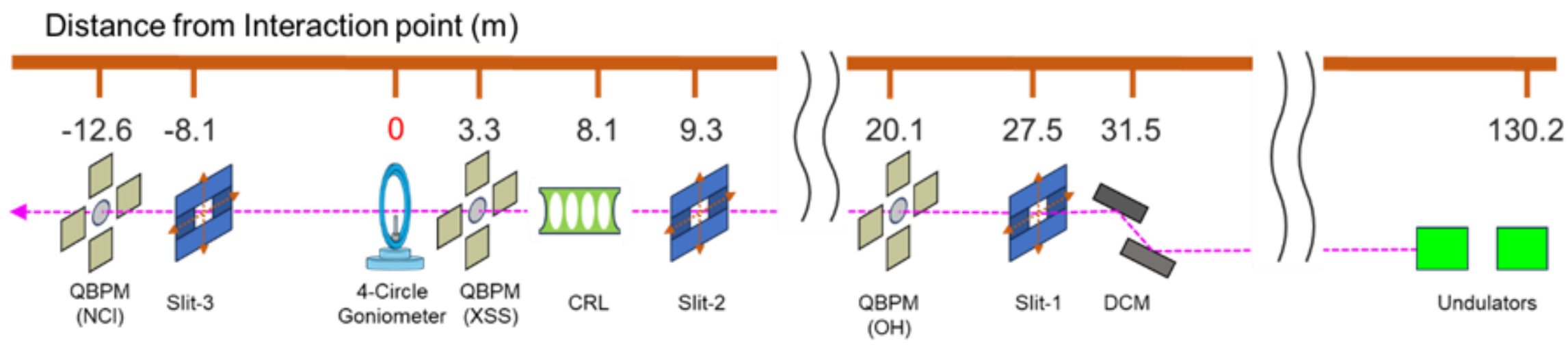

Figure S3 Schematic layout of the XSS beamline at PAL-XFEL. Component positions are given as distances from the IP (z = 0 m). Key components: Slit-3 (−8.1 m), CRL (8.1 m), Slit-2 (9.3 m), Slit-1 (27.5 m), DCM (31.5 m), and the PAL-XFEL undulators (130.2 m). The physical X-ray source is taken as the exit of the second-to-last undulator segment at 130.2 m upstream of the IP. The vertically deflecting Si(111) DCM acts as a virtual source in the vertical plane, giving rise to the observed source astigmatism between the horizontal and vertical apparent source distances.

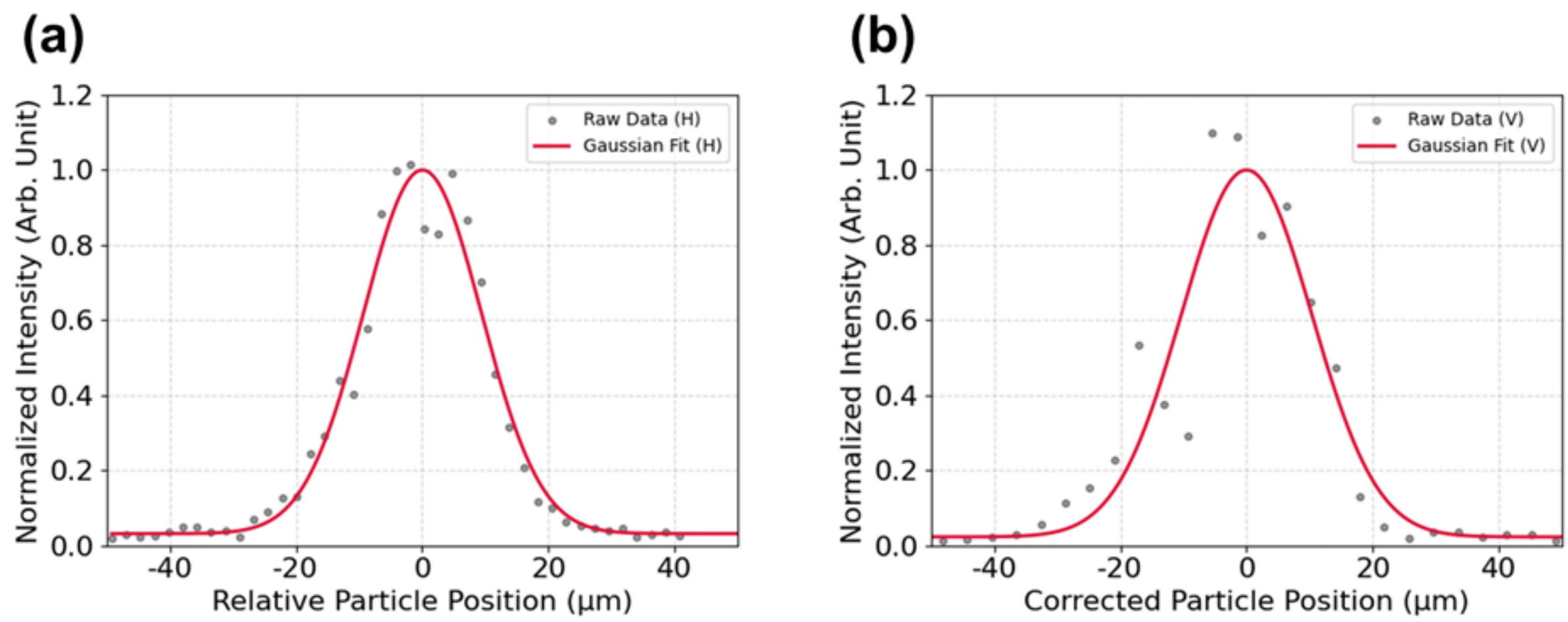

Figure S4 Characterization of the focused X-ray beam profile at the IP. (a) Horizontal and (b) Vertical beam profiles obtained by scanning a single Au nanocrystal across the focal plane (gray dots). The red lines represent gaussian fits used to determine the beam dimensions. For the vertical profile in (b), the positions are deduced by accounting for the 13.4° tilt of the sample in the Bragg reflection geometry.

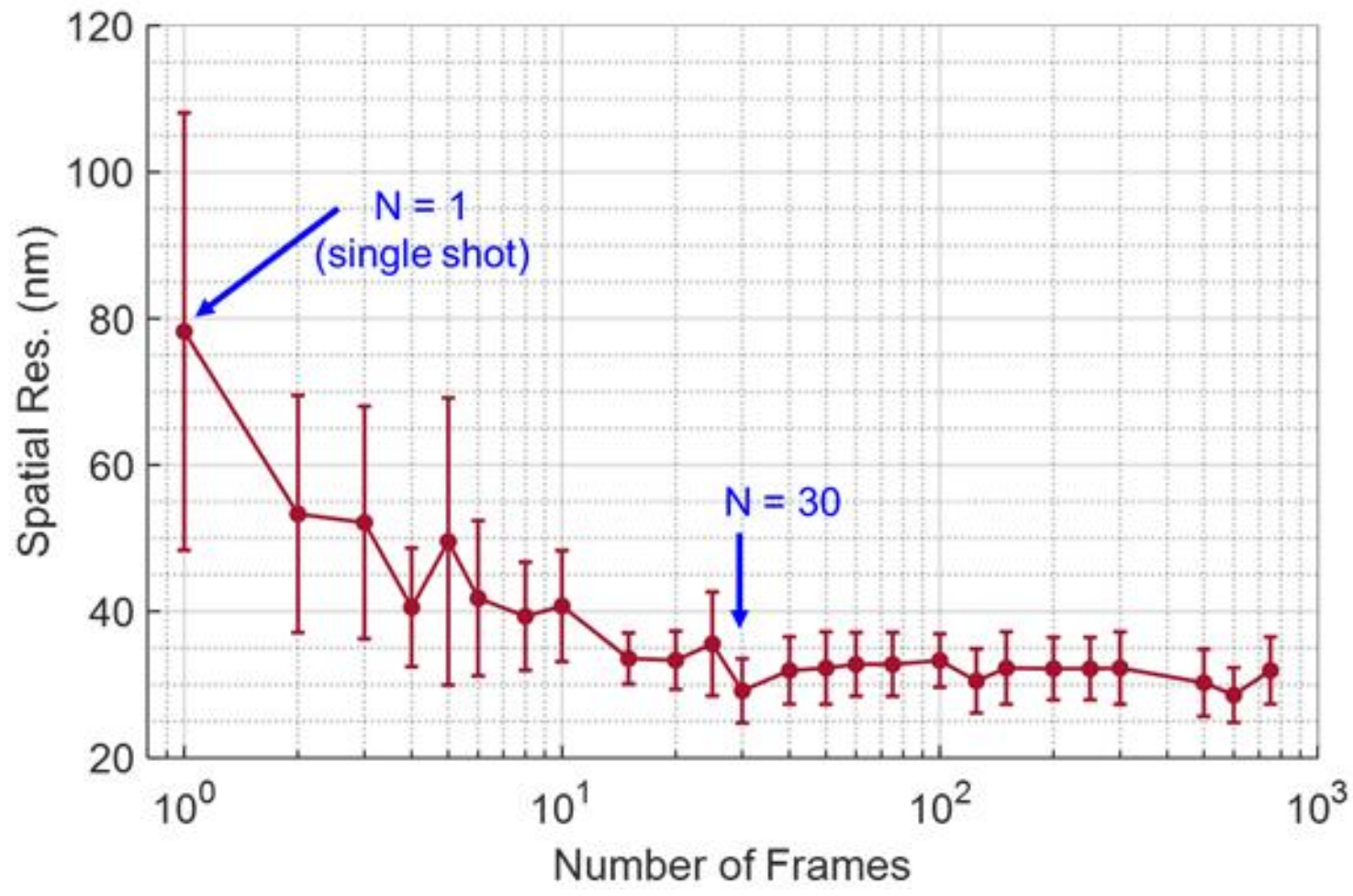

Figure S5 Optimization of the shot accumulation number (N) for spatial resolution convergence. The data points and error bars represent the mean and standard deviation of the ten resolution estimates at each N.
